# Reduced FRG1 expression promotes prostate cancer progression and affects prostate cancer cell migration and invasion

**DOI:** 10.1186/s12885-019-5509-4

**Published:** 2019-04-11

**Authors:** Ankit Tiwari, Bratati Mukherjee, Md. Khurshidul Hassan, Niharika Pattanaik, Archita Mohanty Jaiswal, Manjusha Dixit

**Affiliations:** 1 School of Biological Sciences, National Institute of Science Education and Research Bhubaneswar, HBNI, PO: Bhimpur-Padanpur, Via: Jatni, Odisha 752050 India; 2 SRL Diagnostics Ltd, Plot 2084, Hall Plot 339/4820, Goutam Nagar Unit no. 28, Bhubaneswar, Odisha 751014 India

**Keywords:** FRG1, Prostate cancer, Immunohistochemistry, Cell migration, Cell invasion, p38 MAPK, GM-CSF, MMP1, PLGF, CXCL1

## Abstract

**Background:**

Prostate cancer is the most common form of cancer in males and accounts for high cancer related deaths. Therapeutic advancement in prostate cancer has not been able to reduce the mortality burden of prostate cancer, which warrants further research. FRG1 which affects angiogenesis and cell migration in Xenopus, can be a potential player in tumorigenesis. In this study, we investigated the role of FRG1 in prostate cancer progression.

**Methods:**

Immunohistochemistry was performed to determine FRG1 expression in patient samples. FRG1 expression perturbation was done to investigate the effect of FRG1 on cell proliferation, migration and invasion, in DU145, PC3 and LNCaP cells. To understand the mechanism, we checked expression of various cytokines and MMPs by q-RT PCR, signaling molecules by western blot, in FRG1 perturbation sets. Results were validated by use of pharmacological inhibitor and activator and, western blot.

**Results:**

In prostate cancer tissue, FRG1 levels were significantly reduced, compared to the uninvolved counterpart. FRG1 expression showed variable effect on PC3 and DU145 cell proliferation. FRG1 levels consistently affected cell migration and invasion, in both DU145 and PC3 cells. Ectopic expression of FRG1 led to significant reduction in cell migration and invasion in both DU145 and PC3 cells, reverse trends were observed with FRG1 knockdown. In androgen receptor positive cell line LNCaP, FRG1 doesn’t affect any of the cell properties. FRG1 knockdown led to significantly enhanced expression of GM-CSF, MMP1, PDGFA and CXCL1, in PC3 cells and, in DU145, it led to higher expression of GM-CSF, MMP1 and PLGF. Interestingly, FRG1 knockdown in both the cell lines led to activation of p38 MAPK. Pharmacological activation of p38 MAPK led to increase in the expression of GM-CSF and PLGF in DU145 whereas in PC3 it led to enhanced expression of GM-CSF, MMP1 and CXCL1. On the other hand, inhibition of p38 MAPK led to reduction in the expression of above mentioned cytokines.

**Conclusion:**

FRG1 expression is reduced in prostate adenocarcinoma tissue. FRG1 expression affects migration and invasion in AR negative prostate cancer cells through known MMPs and cytokines, which may be mediated primarily via p38 MAPK activation.

**Electronic supplementary material:**

The online version of this article (10.1186/s12885-019-5509-4) contains supplementary material, which is available to authorized users.

## Background

Prostate cancer is the most common form of cancer in men, and is the second and third most common cause of cancer-related death of men, in the US and Europe, respectively [1, 2]. It is a heterogeneous disease in early stages, which requires rigorous stratification, so that the progression to the advanced stage could be predicted more accurately [3]. Advances are being made in the treatment and etiological understanding of prostate cancer. Still the abundance of resistant tumor types and the burden of prostate cancer related death, poses a question regarding better etiological understanding of the disease [4]. Consequently, the search for novel regulators and molecular mechanism, associated with prostate cancer, is of the utmost significance.

To enhance the understanding about additional players in prostate cancer, FRG1 can be a good candidate. FRG1 is a candidate gene for Facioscapulohumeral muscular dystrophy (FSHD) [5] but it has also been shown to affect vasculature [6]. Functional domain analysis of FRG1 revealed that it consists of a fascin like domain. Fascin is an actin bundling protein which is known to be involved in tumor progression [7]. FRG1 is also essential for differentiation of amleoblasts, odontoblasts and matrix formation, associating it with BMP4 which is well known tumorigenesis regulator [8]. Above mentioned studies provide indications of possible involvement of FRG1 in tumorigenesis. Oncomine datamining, in our previous work, showed the reduced expression of FRG1 in more studies, which includes prostate cancer. Additionally, Kaplan Meier plotter analysis revealed that low FRG1 expression was associated with poor prognosis in overall survival of patients, in lung cancer and in gastric cancer [9]. Somatic mutation analysis shows 0.24% frequency in COSMIC [10], 1.8% in cBioPortal [11] [12], and 5.37% in TCGA databases, respectively. Couple of recent studies have reported mutations in FRG1 gene in cancer patients [13] [14].

On the whole, all above mentioned facts strongly support towards the possible role of FRG1 in prostate cancer, which is completely unexplored. Therefore, we intend to understand the etiological function of FRG1 in prostate cancer. In this study we used three different prostate cancer lines to establish role of FRG1 with respect to androgen receptor status and varying invasiveness. DU145 and PC3 cell lines are androgen receptor (AR) negative and are more invasive, on the contrary, LNCaP is AR positive and less invasive. We perturbed the expression levels of FRG1 and checked the effect on various cell properties. We used DU145 and PC3 cell line, in which we found positive effect of FRG1 expression level on cell properties, for mechanistic studies.

## Methods

### Patients and tissue microarray

Study included archival formalin-fixed, paraffin-embedded (FFPE) blocks from patients with acinar adenocarcinoma of prostate (*N* = 20), who underwent needle core biopsy of prostate from 2014 to 2015. FFPE blocks were obtained from tissue archives of SRL Bhubaneswar, at Kalinga Hospital Pvt. Limited Bhubaneswar, India. Patient who had undergone treatment prior to biopsy were excluded from our study. All the cases were reviewed by two independent pathologists N.P. and A.M.J. The clinicopathological features for cases were identified prior to selection of cases. The study was approved (BioEthics # MD-1) by institutional ethics committee, National Institute of Science Education and Research (NISER), Bhubaneswar, India. Further, to increase sample size, tissue array consisting of 90 cores of prostate adenocarcinoma along with paired uninvolved adjacent tissue was procured from Biomax (USA).

### Immunohistochemistry

The tumor tissue was fixed in 10% buffered formalin, embedded in paraffin, and serially sectioned at 4 μm thickness. Sections were deparaffinized and rehydrated. Heat induced epitope retrieval was done using microwave in Envision target retrieval solution High pH (Dako, USA). Immunohistochemistry procedure was followed as mentioned in Dako Envision Plus kit manual (Dako). FRG1 antibody was diluted at 1:100 for immunohistochemistry (IHC). HeLa cell block was stained, as control for FRG1 antibody. The pathologist blinded to patient’s background, scored the staining of FRG1 and micro-vessel density (MVD) count.

The expression levels of FRG1 protein in the cytoplasm and nucleus of tumor cells were scored as defined by Immunoreactive score, described by Fedchenko et al. [15]. MVD count was assessed as per Wiedner et al. [16]. Imaging and scoring were done using upright light microscope (CX31, Olympus, Japan), using 10X and 40X objective lenses.

### Plasmids, cell culture, transfection, reagents

FRG1 expression vector (pCMV6.Xl5-FRG1) and knockdown vector (pLKO.1- FRG1sh) along with their controls were procured from Origene (USA) and Sigma (USA), respectively. Plasmids were purified using Plasmid midi kit (Qiagen) according to manufacturer’s guidelines.

PC3 cells (ATCC® CRL-1435™) were obtained from National Centre for Cell Science (Pune, India) in 2015 and, were grown in RPMI1640 (HiMedia Labs, India) with 10% FBS (Pan Biotech, Germany). DU145 (ATCC® HTB-81™), a gift from Dr. Rajeeb Swain’s Lab (Institute of Life sciences, Bhubaneswar, India) received in 2015, was grown in DMEM (Pan Biotech) with 10% FBS. LNCaP cells (ATCC® CRL-1740™) were purchased from National Centre for Cell Science (Pune, India) in 2019. LNCaP cell line was found authentic, using AmpFLSTR Identifiler Plus PCR Amplification kit (Applied Biosystems, USA). DU145 and PC3 cell lines were found authentic, using AmpFLSTR Identifiler PCR Amplification kit (Applied Biosystems, USA). All the cell lines were tested for mycoplasma contamination (Lonza, MycoAlert Mycoplasma Assay Kit) and found free from it.

DU145 and PC3 cells were grown in DMEM (Pan Biotech) with 10% FBS. LNCaP cells were grown in RPMI (Himedia) with 15% FBS. All the cell transfections were carried out using Lipofectamine 3000 (Invitrogen, USA), as per manufacturer’s protocol. Cell lysate for FRG1 over-expression and knockdown conditions were prepared after 48 h and 72 h of transfection respectively, in both the cell lines. The pharmacological activator and inhibitor of p38 MAPK, Anisomycin (Calbiochem, USA) and SB203580 (Merck, USA) respectively, were used to modulate p38 MAPK activity in prostate cancer cells treated with FRG1 knockdown and scrambled sequence vectors. DU145 and PC3 cells were treated with 0.4 μg/ml Anisomycin (for 2 h and 6 h) and 0.5 μM SB203580 (for 2 h) and harvested for western blotting. Similarly, cells were harvested 8 h post-treatment for total RNA extraction.

### Western blot

For cell lysate preparation, cells were washed with ice-cold phosphate-buffered saline (PBS) and lysed in ice-cold RIPA buffer (Thermo Fisher Scientific, USA), with added protease inhibitor (Sigma) and phosphatase inhibitor (Sigma). Protein quantification of cell lysate was done using BCA reagent (Thermo Fisher Scientific). 30 μg of protein sample was mixed with equal volume of 2X Laemilli buffer and boiled at 95 °C for 10 min. The lysates were separated on 10% sodium dodecyl sulfate–polyacrylamide gel electrophoresis (SDS-PAGE). The proteins, on the gels, were transferred to polyvinylidene fluoride (PVDF) membrane (Millipore, USA). The blots were probed with specific antibodies for FRG1 (1:1000 dilution) (Novus Biologicals, USA), ERK (1:1000) (Sigma), phospho ERK (1:1000), p38 MAPK (1:1000) (Cell Signaling Technology, USA), phospho p38 MAPK (1:1000) (Cell Signaling Technology) followed by HRP tagged anti-mouse IgG secondary antibody (Thermo Fisher Scientific) for FRG1 and, HRP tagged anti rabbit IgG secondary antibody for ERK, phospho ERK, p38 MAPK and, phospho p38 MAPK. The labeled bands were subsequently detected by chemiluminescence. For each sample, band intensities were normalized to GAPDH (1:10000) (Sigma).

### Cell proliferation assay

LNCaP, PC3 and DU145 cells (0.1 × 10^4^) were seeded in a 96 well plate. Cells were transfected with FRG1 expression and silencing vectors along with their respective controls. Transfected cells were grown for 96 h. Afterwards culture medium was replaced with 100 μl fresh medium and, 20 μl of Cell titer 96 AQueous one solution reagent (Promega, USA) was added. Plates were incubated at 37 °C for 1 h. Absorbance was recorded at 490 nm wavelength using Varioskan multimode reader (Thermo Fisher Scientific).

### Wound healing assay

PC3 and DU145 cells (0.25 × 10^6^) were seeded into 6 well plates. Transfection was done with FRG1 expression and silencing vectors along their respective controls. Cells were allowed to grow till 100% confluency. Scratch was made in the plate using a P200 pipette tip. Images were collected at 0 h and 24 h for PC3 and 0 h and 48 h for DU145, under inverted microscope (Ziess, Germany). Cell migration was analyzed using NIH imageJ software.

### Transwell migration assay

Transwell migration assay was performed using Millipore transwell chambers (8 μm pore size, Millipore). PC3, DU145 and LNCaP cells (2 × 10^4^ in each well), transfected with FRG1 expression vector and knockdown vector with their respective vector controls, were seeded in the upper chambers of the 12 well plate (Corning, USA) in 500 μl serum-free medium. The lower chambers were filled with 1 ml complete medium. The chamber was incubated at 37 °C for 24 h (60 h for LNCaP). At the end of incubation, the cells in the upper surface of the membrane were removed with a cotton swab. Cells in lower chamber were fixed with methanol and stained with Giemsa (HiMedia Labs). The images were taken with inverted microscope (CX41, Olympus) and analyzed using NIH imageJ software.

### Cell invasion assay

Matrigel invasion assay and analysis was done as mentioned in transwell migration assay section. With an exception, in cell invasion assay, Millipore transwell chambers (8 μm pore size, Millipore) were coated with 1 mg/ml growth factor reduced matrigel (Corning) prior to the assay.

### Q-RT-PCR

Quantitative real time PCR was done to find out the effect of FRG1 expression on the expression of certain MMPs and cytokines. Three biological replicates of PC3 and DU145 cells with ectopic FRG1 expression and FRG1 knockdown along with their controls, were prepared. Total RNA was isolated with RNeasy mini kit (Qiagen) according to the manufacturers’ protocol, concentration was measured using a NanoDrop 2000 spectrophotometer (Thermo Fisher Scientific). The RNA was converted to cDNA using the Superscript IV reverse transcriptase (Invitrogen). RNA extraction for cells treated with Anisomycin and SB203580 was performed after 8 h post treatment, using RNeasy minikit (Qiagen) followed by cDNA synthesis, using verso cDNA synthesis kit (Thermo Fisher Scientific).

qRT-PCR was performed for cytokines and MMPs, for which primers are mentioned in the (Additional file 1: Table S1), using the Fast start Universal SYBR Green master mix (Roche, Switzerland) with an ABI 7500 system (Applied Biosystems, USA). All the reactions were done in triplicate, using GAPDH as internal control.

### Statistical analysis

Statistical analysis was performed using Graphpad Prism 7.00, Microsoft Excel and SPSS. For comparison of FRG1 expression in tumor versus uninvolved tissue, 2-tailed chi square test was applied to determine the significance. For chi square analysis IRS scores were recoded into negative (0–1), weak (2–3), moderate (4–8) and strongly positive (9–12), as described by Fedchenko et al. [15]. Fisher’s exact test was carried out to associate negative (0–1) IRS score with tumor. Spearman correlation analysis was used to find out association of FRG1 IRS score with Gleason score in tumor samples and, with MVD. Student’s t-test (2-tailed, unpaired) was performed to identify statistical significance in cell based assay data. We used SPSS (version 17) General linear model (GLM), univariate analysis, blocked by cell type and AR status, to find out the effect of FRG1 expression level, using combined data of all three cell types, on cell proliferation, migration and invasion. Regression and ANCOVA analysis was used to determine interaction of wound healing, migration and invasion. Q-Q plots were used to ascertain the normal distribution of data. *p* value ≤0.05 was considered to be significant in all the tests.

## Results

### FRG1 levels in prostate adenocarcinoma

**Fig. 1:**
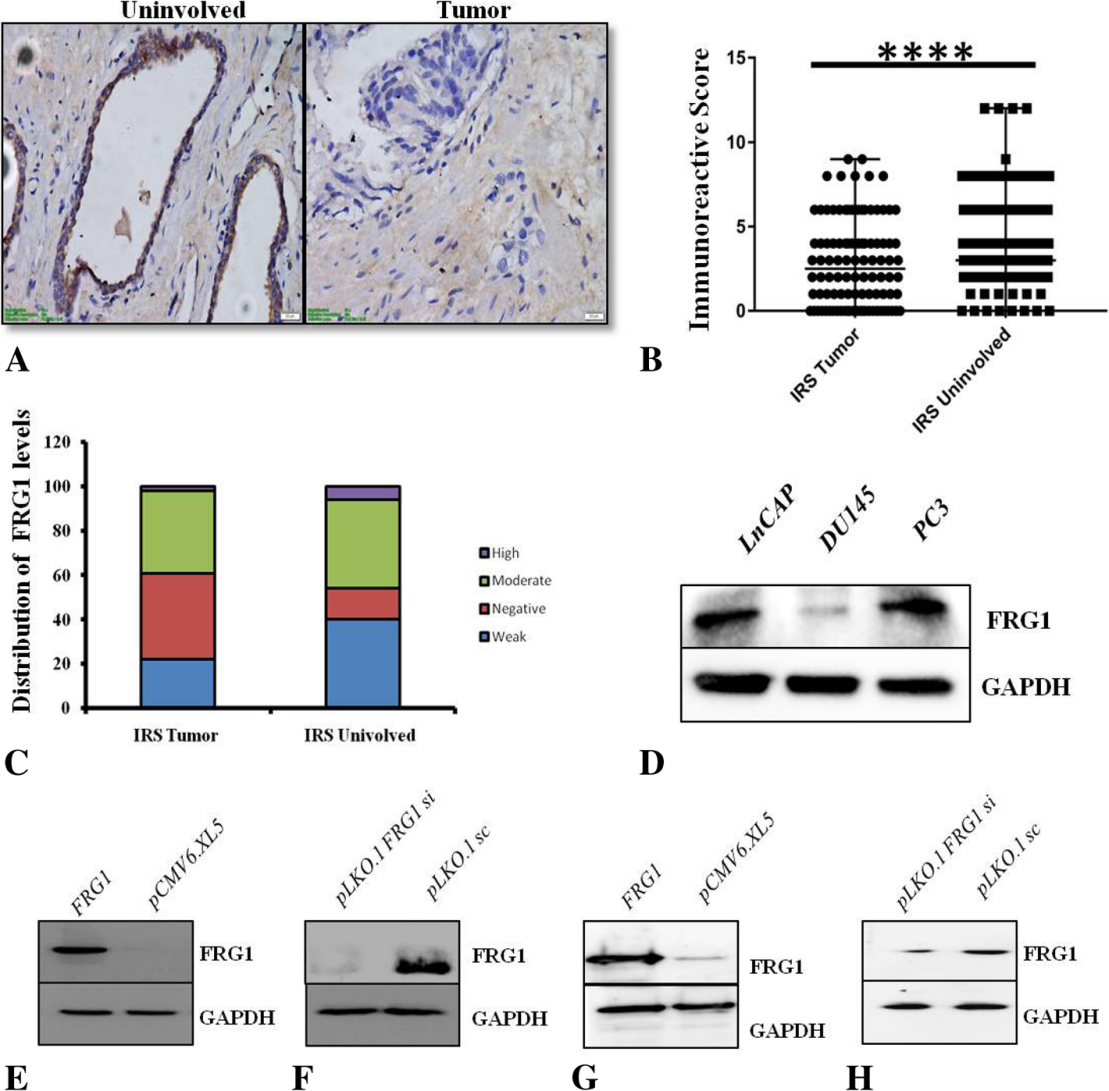
FRG1 expression levels in prostate tumor and cell lines: **a**. Representative images of tumor and uninvolved tissues of prostate, as seen in first (uninvolved) and second (tumor) column from left. **b**. Comparison of IRS between tumor and uninvolved tissue. Graph shows that the reduction of IRS in tumor tissue (*N* = 100) compared to uninvolved adjacent tissue (*N* = 100) was significant (chi square test, 2 tailed, df-5, *p* value < 0.0005). Median IRS score for FRG1 in tumor is 2.5 compared to adjacent uninvolved tissue, which is 3.5. **c**. Distribution of staining pattern for FRG1 in the prostate tumor (*N* = 100) and uninvolved tissue (*N* = 100). **d**. FRG1 expression levels in three different prostate cancer cells, western blot panel shows comparatively higher levels of FRG1 in PC3 and LNCaP than DU145 cells. **e**. Western blot to confirm ectopic expression of FRG1 in DU145 cells. **f**. Western blot showing reduced FRG1 levels after RNAi silencing in DU145. **g**. Western blot image validating ectopic expression of FRG1 levels in PC3 cells. **h**. Reduction in FRG1 levels in FRG1 silenced PC3 cells confirmed by western blot. **** represents *p* value < 0.0005, N represents number of patient samples

FRG1 expression was analyzed in prostate cancer by immunohistochemistry in 20 needle core biopsies along with tissue array, consisting of 180 cores (including 90 paired tumor and uninvolved tissue). Out of 20 needle core biopsies, uninvolved prostate tissue was present in 10 biopsies. For prostate cancer samples, cohort information has been provided in (Additional file 2: Table S2). Figure 1a shows strong FRG1 staining in control tissue, compared to tumor tissue. The staining pattern revealed significant reduction of FRG1 expression levels in tumor cells, compared to uninvolved secretory ductal epithelial cells of prostate. Immunoreactive score (IRS), quantified for the staining pattern, revealed that 52 out of 100 cases (*p* value < 0.0005) had reduced FRG1 expression in tumor tissue (Fig. 1b). FRG1 staining was negative in 39% of tumor tissue compared to 14% of uninvolved tissue. Fisher’s exact test (2-sided, df = 1) showed significant (*p* < 0.0001) association of negative IRS score with tumor. Staining pattern of FRG1 was mostly moderate to weak in both tumor and uninvolved tissue, with 22% weak in tumor to 40% weak expression in uninvolved tissue. Moderate staining was distributed evenly with 37% tumor cases showing moderate staining, compared to 40% cases of uninvolved tissue. 6% uninvolved tissue had high FRG1 staining compared to 2% of tumor tissue (Fig. 1c). FRG1 expression (IRS) showed significant negative correlation (Spearman correlation, 2-tailed, r^2^ -0.285, *p* value < 0.005) with tumor grade (Gleason score) (Additional file 3).

Further, to understand the effect of FRG1 expression on tumor angiogenesis, correlation analysis was done for FRG1 IRS and MVD. No significant correlation (Spearman correlation, 2-tailed) could be derived between FRG1 protein expression levels and MVD (*p* value > 0.05, r^2^ 0.105) (Additional file 3). Overall, patient IHC data revealed that FRG1 expression is reduced in tumor tissue but does not correlate with MVD count.

### FRG1 expression doesn’t correlate with AR status in prostate cancer cell lines

To find out if there is any prostate cancer cell line specific expression pattern of FRG1, the endogenous FRG1 expression levels were determined in PC3, LNCaP, and DU145 cells. PC3 and LNCaP cells had higher FRG1 expression compared to DU145 (Fig. 1d). As PC3 and DU145 are androgen receptor negative cells and LNCaP is androgen receptor positive, FRG1 expression cannot be correlated with the presence of receptor.

### Varying effect of FRG1 on proliferation of AR negative prostate cancer cells

**Fig. 2:**
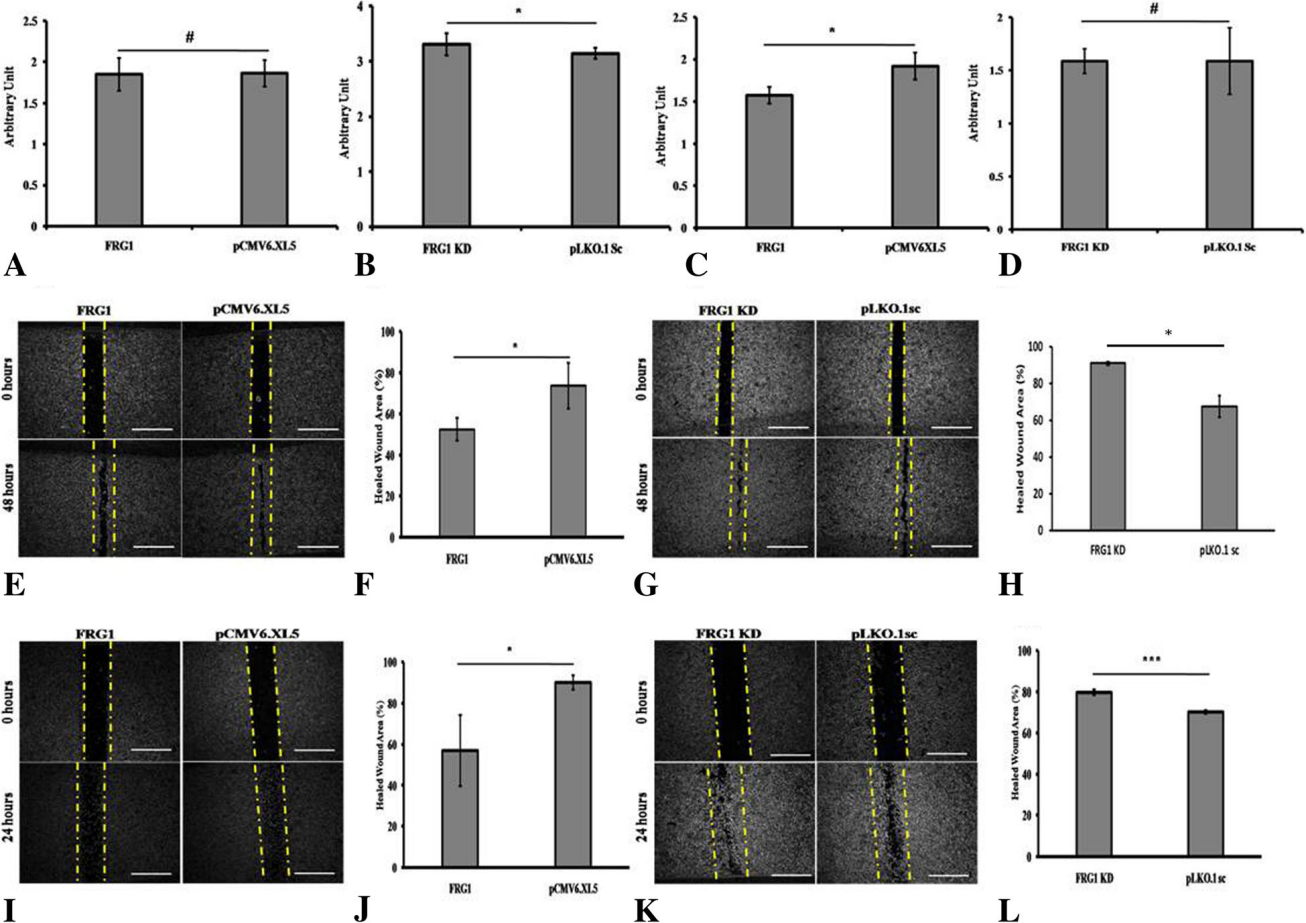
Effect of FRG1 expression on cell proliferation and scratch wound healing: **a**. Ectopic expression of FRG1 has no significant (*N* = 5, 2-tailed unpaired t-test, *p* value > 0.05) effect on cell proliferation in DU145, compared to empty vector control. **b**. Measurement of cell proliferation in DU145, knockdown of FRG1 showing significant increase in cell proliferation (*N* = 5, 2-tailed unpaired t-test, *p* value < 0.05), compared to scrambled vector control. **c**. Measurement of cell proliferation in PC3 with ectopic expression of FRG1, compared to empty vector control, showing significant reduction (*N* = 5, 2-tailed unpaired t-test, *p* value < 0.05). **d**. Quantitation of cell proliferation in PC3 with knockdown of FRG1, compared to scrambled vector control, showing no significant (*N* = 5, 2-tailed unpaired t-test, *p* value > 0.05) effect. **e**. Representative images of scratch wound healing assay of DU145 cells, with ectopic expression of FRG1 and respective vector control. **f**. Graph of scratch wound healing assay of DU145 cells with ectopic expression of FRG1, showing 52% reduced wound area, compared to empty vector control with 74% reduced wound area (*N* = 3, 2-tailed unpaired t-test, *p* value < 0.05). **g**. Representative images of scratch wound healing assay of DU145 with FRG1 knockdown and respective scrambled vector control. **h**. Graph of scratch wound healing assay of DU145 cells with FRG1 knockdown showing 91% reduced wound area, compared to scrambled vector control with 68% reduced wound area (*N* = 3, 2-tailed unpaired t-test, *p* value < 0.05). **i**. Representative images of scratch wound healing assay of PC3 cells with ectopic expression of FRG1 and respective vector control. **j**. Graph of scratch wound healing assay of PC3 cells with ectopic expression of FRG1, showing 57% reduced wound area, compared to empty vector control with 90% reduced wound are (*N* = 3, 2-tailed unpaired t-test, *p* value < 0.05). **k**. Representative images of scratch wound healing assay of PC3 with FRG1 knockdown and respective scrambled vector control. **l**. Graph of scratch wound healing assay of PC3 cells with FRG1 knockdown, showing 80% reduced wound area, compared to scrambled vector control with 70% reduced wound area (*N* = 3, 2-tailed unpaired t-test, *p* value < 0.005). # represents *p* value > 0.05, * represents *p* value ≤0.05, ** represents *p* value < 0.01, *** represents *p* value < 0.005, N represents experiment replicates

To find out if FRG1 has any direct effect on proliferation, we prepared DU145 and PC3 cells with ectopic expression of FRG1 and with depletion of FRG1, along with their controls (Fig. 1e-h). Cell proliferation assay revealed that FRG1 over-expression had no significant effect on proliferation of DU145 cells (Fig. 2a) but FRG1 knockdown led to significant increase in cell proliferation (Fig. 2b). PC3 cells had significantly reduced proliferation in response to ectopic expression of FRG1 (Fig. 2c) but no significant effect was observed on cell proliferation of PC3 cells with FRG1 knockdown (Fig. 2d).

### FRG1 affects motility and invasiveness in AR negative prostate cancer cells

Enhanced cell motility and invasiveness are important features of tumor progression. Therefore, to investigate the role of FRG1 in cell migration and invasion we performed scratch wound healing, transwell cell migration and matrigel invasion assays.

Wound healing was significantly (*p* value < 0.05) reduced in cells ectopically expressing FRG1, with 52% area healed in FRG1 expression set compared to 74% of empty vector, in DU145. In PC3 cell line, FRG1 expression led to 57% area being healed, compared to 90% of empty vector set (*p* value < 0.05) (Fig. 2e-f, i-j, respectively). To confirm the findings, scratch wound healing assay was done in FRG1 knockdown set. Increased wound healing in FRG1 knockdown set was observed, compared to scrambled control vector set (91% vs. 68% respectively, *p* value < 0.05) in DU145 (Fig. 2g-h). In PC3 cells, FRG1 knockdown led to 80% reduced wound area compared to 70% of scrambled vector set (*p* value < 0.005) (Fig. 2k-l).

**Fig. 3:**
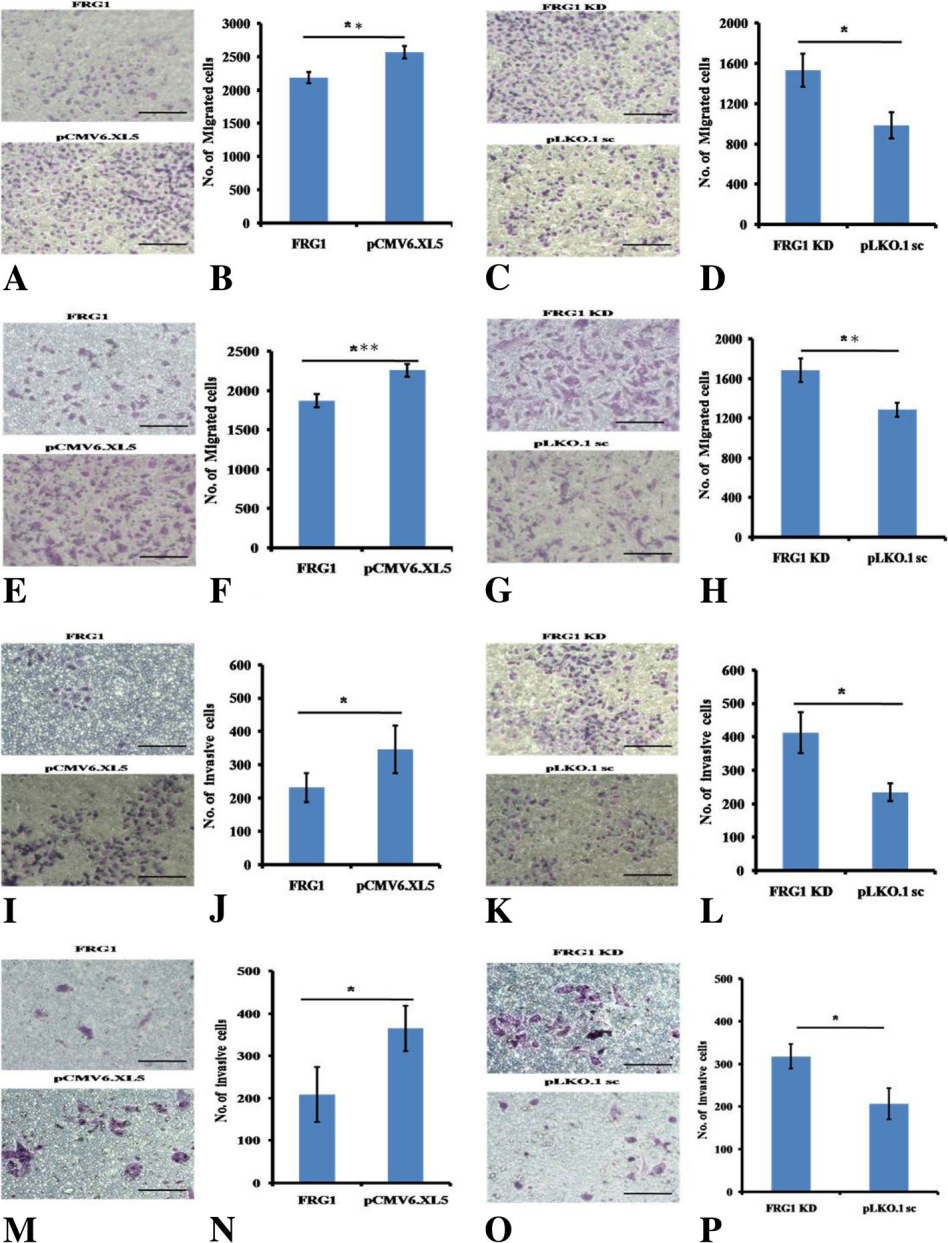
Effect of FRG1 expression on transwell migration and invasion: **a**. Representative images of transwell migration assay of DU145 cells with ectopic expression of FRG1 and respective vector control. **b**. Graphical representation of transwell migration assay of DU145 cells with ectopic expression of FRG1 (2183 ± 84), showing reduction in transwell migration, compared to empty vector control (2565 ± 94) (*N* = 3, 2-tailed unpaired t-test, *p* value < 0.01). **c**. Representative images of transwell migration assay of DU145 cells with FRG1 knockdown and respective scrambled vector control. **d**. Graph of transwell migration assay of DU145 cells with FRG1 knockdown (1532 ± 165), showing enhanced transwell migration, compared to scrambled vector control (987 ± 130) (*N* = 3, 2-tailed unpaired t-test, *p* value < 0.05). **e**. Representative images of transwell migration assay of PC3 cells with ectopic expression of FRG1 and respective vector control. **f**. Graphical representation of transwell migration assay of PC3 with ectopic expression of FRG1 (1869 ± 86), showing reduction (*N* = 3, 2-tailed unpaired t-test, *p* value < 0.005) in transwell migration, compared to empty vector control (2256 ± 81). **g**. Representative images of transwell migration assay of PC3 cells with FRG1 knockdown and respective scrambled vector control. **h**. Graphical representation of transwell migration assay of PC3 cells with FRG1 knockdown (1685 ± 120), showing enhanced (*N* = 3, 2-tailed unpaired t-test, *p* value < 0.01) transwell migration, compared to scrambled vector (1285 ± 71). **i**. Representative images of matrigel invasion assay of DU145 cells with ectopic expression of FRG1 and respective vector control. **j**. Graphical representation of matrigel invasion assay of DU145 cells with ectopic expression of FRG1 (231 ± 43) compared to empty vector control (357 ± 60) (*N* = 3, 2-tailed unpaired t-test, *p* value < 0.05). **k**. Representative images of matrigel invasion assay of DU145 with FRG1 knockdown and respective scrambled vector control. **l**. Representative graph of matrigel invasion assay of DU145 cells with FRG1 knockdown (412 ± 62) compared to scrambled vector control (234 ± 27) (*N* = 3, 2-tailed unpaired t-test, *p* value < 0.05). **m**. Representative images of matrigel invasion assay of PC3 cells with ectopic expression of FRG1 and respective vector control. **n**. Representative graph of matrigel invasion assay of PC3 cells with ectopic expression of FRG1 (208 ± 65) compared to empty vector control (365 ± 53) (*N* = 3, 2-tailed unpaired t-test, *p* value < 0.05). **o**. Representative images of matrigel invasion assay of PC3 with FRG1 knockdown and respective scrambled vector control. **p**. Representative graph of matrigel invasion assay of PC3 cells with FRG1 knockdown (318 ± 29) compared to scrambled vector control (207 ± 37) (*N* = 3, 2-tailed unpaired t-test, *p* value < 0.05). * represents *p* value ≤0.05, ** represents *p* value < 0.01, *** represents *p* value < 0.005, N represents experiment replicates

**Fig. 4:**
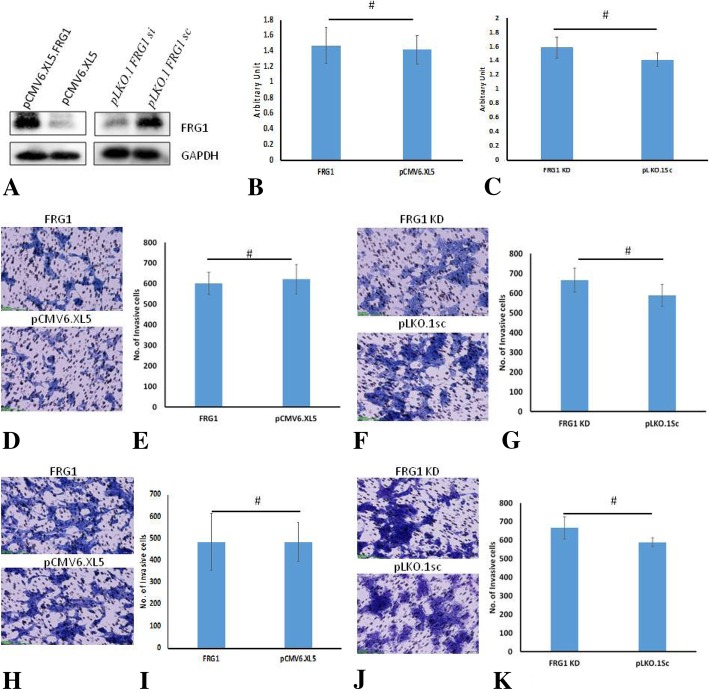
Effect of FRG1 expression on LNCaP cell properties. **a**. Western blot to confirm ectopic expression of FRG1 in LNCaP cells. Western blot to confirm depletion of FRG1 expression in LNCaP cells after RNAi silencing. **b**. Graphical representation of LNCaP cell proliferation assay showing that ectopic expression of FRG1 has no significant (*N* = 5, 2-tailed unpaired t-test, *p* value > 0.05) effect on cell proliferation, compared to empty vector control (pCMV6.XL5). **c**. Graph showing that knockdown of FRG1 (FRG1 KD) doesn’t change LNCaP cells proliferation significantly (*N* = 5, 2-tailed unpaired t-test, *p* value > 0.05), compared to control (pLKO.1sc). **d**. Representative images of transwell migration assay of LNCaP cells with ectopic expression of FRG1 (FRG1) and respective vector control (pCMV6.XL5). **e**. Graphical representation of transwell migration assay of LNCaP cells with ectopic expression of FRG1 (601 ± 54), showing no change in transwell migration, compared to empty vector control (622 ± 71) (*N* = 3, 2-tailed unpaired t-test, *p* value > 0.05). **f**. Representative images of transwell migration assay of LNCaP cells with FRG1 knockdown (FRG1 KD) and respective scrambled vector control (pLKO.1sc). **g**. Graph of transwell migration assay of LNCaP cells with FRG1 knockdown (645 ± 61), showing no change in transwell migration, compared to scrambled vector control (550 ± 55) (*N* = 3, 2-tailed unpaired t-test, *p* value > 0.05). **h**. Representative images of matrigel invasion assay of LNCaP cells with ectopic expression of FRG1 and respective vector control. **i**. Graphical representation of matrigel invasion assay of LNCaP cells with ectopic expression of FRG1 (483 ± 130) compared to empty vector control (484 ± 89) (*N* = 3, 2-tailed unpaired t-test, *p* value > 0.05). **j**. Representative images of matrigel invasion assay of LNCaP cells with FRG1 knockdown (FRG1 KD) and respective scrambled vector control (pLKO.1sc). **k**. Representative graph of matrigel invasion assay of LNCaP cells with FRG1 knockdown (666 ± 60) compared to scrambled vector control (589 ± 24) (N = 3, 2-tailed unpaired t-test, *p* value > 0.05). # represents *p* value > 0.05, N represents experiment replicates

Further support was provided by transwell cell migration data, which was decreased in cells ectopically expressing FRG1, compared to empty vector control, in both DU145 (*p* value < 0.01) and PC3 (*p* value < 0.005) cell lines (Figs. 3a-b and 4e-f, respectively). This observation was reversed when FRG1 expression was silenced, in both DU145 (*p* value < 0.05) and PC3 (*p* value < 0.01) (Figs. 3c-d and 4g-h, respectively). Ectopic expression of FRG1 led to significant reduction in cell invasion in both DU145 (*p* value < 0.05) and PC3 (*p* value < 0.05) cells (Figs. 3i-j and 4m-n, respectively). FRG1 knockdown had opposite effect on cell invasion, as FRG1 knockdown led to increase in cell invasion in both DU145 (*p* value < 0.05) and PC3 (*p* value < 0.05) cells (Figs. 3k-l and 4o-p, respectively). These results clearly indicate that FRG1 reduces cell migration and invasion in prostate cancer cells *in vitro*.

### No effect of FRG1 expression on cell properties of AR positive cell line

To figure out if the effect of FRG1 expression on prostate cancer cells is irrespective of androgen receptor status, we used LNCaP cell lines with ectopic expression of FRG1 and depletion of FRG1 along with their controls (Fig. 4a), for cell based assays. We found that FRG1 ectopic expression didn’t change proliferation (Fig. 4b), migration (Fig. 4d-e) or invasion (Fig. 4h-i) properties of LNCaP cells significantly (*p* value > 0.05), compared to its control. Similarly, depletion of FRG1 expression didn’t change proliferation (Fig. 4c), migration (Fig. 4f-g) or invasion (Fig. 4j-k) properties of LNCaP cells (*p* value > 0.05). These results indicate that the effect of FRG1 expression is cell line specific and might be dependent upon androgen receptor status.

### Collectively three cell lines show significant effect of FRG1 expression level on cell migration and invasion

To figure out the general effect of FRG1 expression of cell properties we combined the data of three cell lines and used GLM, univariate test and blocked by cell type and AR status. We found that FRG1 expression doesn’t affect cell proliferation significantly (*p* value > 0.05). However, ectopic expression affected the migration (*p* value < 0.0005) and invasion (*p* value < 0.05) of prostate cancer cell lines significantly. Likewise, knock down of FRG1 led to significant difference in rate of migration (*p* value < 0.0001) and invasion (*p* value < 0.0001). We didn’t find any interaction between migration (wound healing and transwell migration) and invasion (*p* value > 0.05).

### FRG1 expression level dictates expression of various cytokines and MMP1

**Fig. 5:**
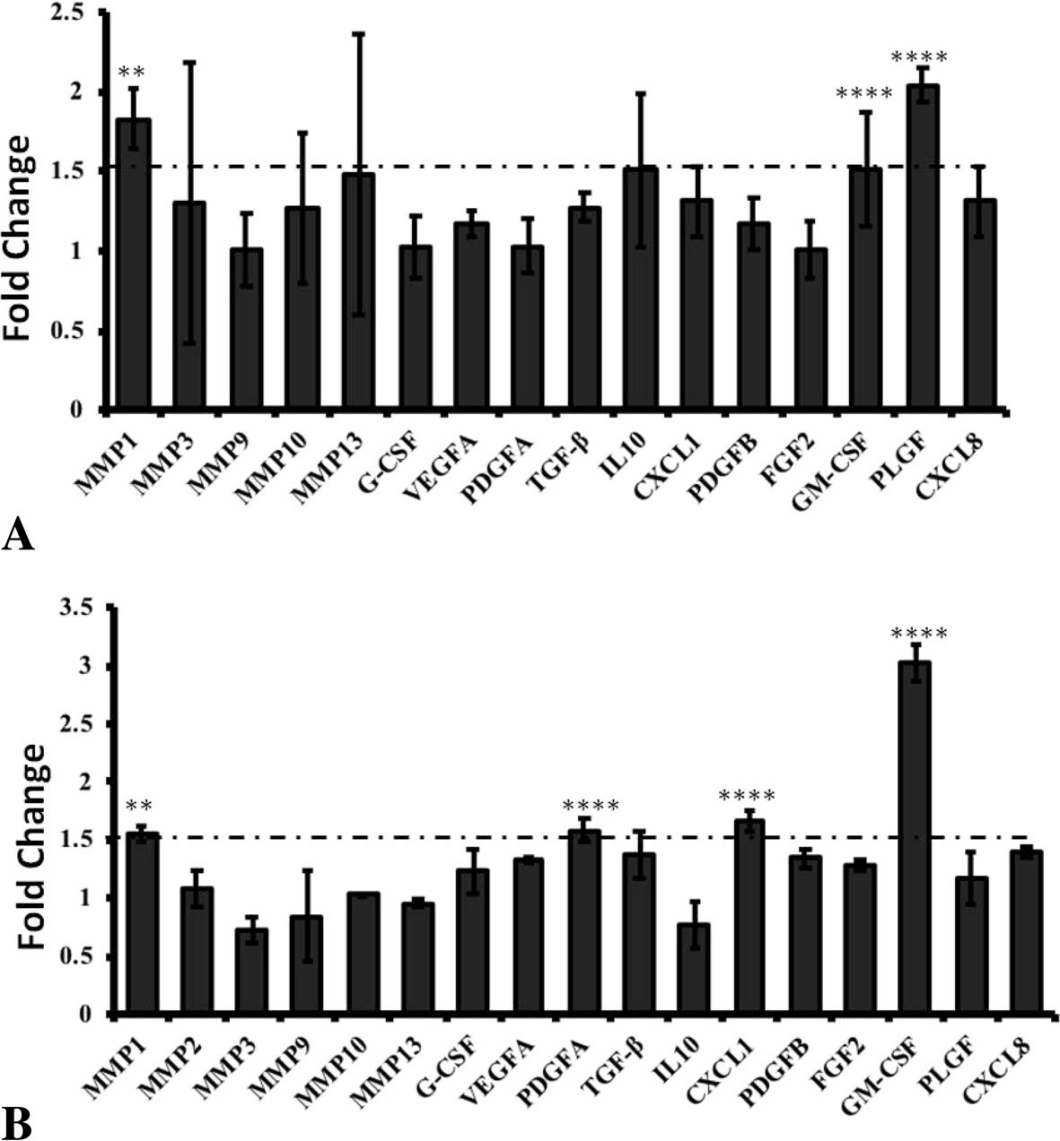
Expression analysis of cytokines and MMPs: **a**. q-RT PCR expression analysis of genes listed in Additional file 1 in DU145 cells, transfected with FRG1 silencing vector compared to scrambled vector control, **b**. q-RT PCR expression data of genes listed in Additional file 1, in PC3 cells with knockdown for FRG1 versus scrambled vector control. In panel A and B, t-test (2 tailed, for unpaired samples) was used for comparison of fold change values between experimental and control group. Each experimental group had three replicates. Dotted line represents 1.5-fold cut off, ** represents *p* value < 0.01, **** represents *p* value < 0.0005

To identify the associated cytokines affected by FRG1 expression, q-RT PCR analysis for 13 cytokines and 7 Matrix metalloproteinases was done (see additional file 1 for primer information). These cytokines have previously been reported to affect cellular phenotypes, i.e. proliferation, migration, invasion and angiogenesis. Ectopic expression of FRG1 led to no significant change in expression of cytokine and matrix metalloproteinases, in both DU145 and PC3 cells (Additional file 4). On the other hand, FRG1 knockdown showed significant change in expression of certain targets in cell line specific manner. FRG1 knockdown in DU145 led to significant increase in expression of GM-CSF (fold change = 1.51, *p* value < 0.0005), PLGF (fold change = 2.04, *p* value < 0.0005) and MMP1 (fold change = 1.83, *p* value < 0.01) (Fig. 5a). By FRG1 silencing in PC3 cells, expression of GM-CSF (fold change = 3.022, *p* value < 0.0005), MMP1 (fold change = 1.56, *p* value < 0.01), PDGFA (fold change = 1.58, *p* value < 0.0005) and CXCL1 (fold Change = 1.67, *p* value < 0.0005) showed significant increase in expression (Fig. 5b). Here we can infer that FRG1 may affect proliferative, migratory and invasiveness properties of cells by modulating expression of above mentioned cytokines and MMPs.

### FRG1 silencing enhances p38 MAPK activation

**Fig. 6:**
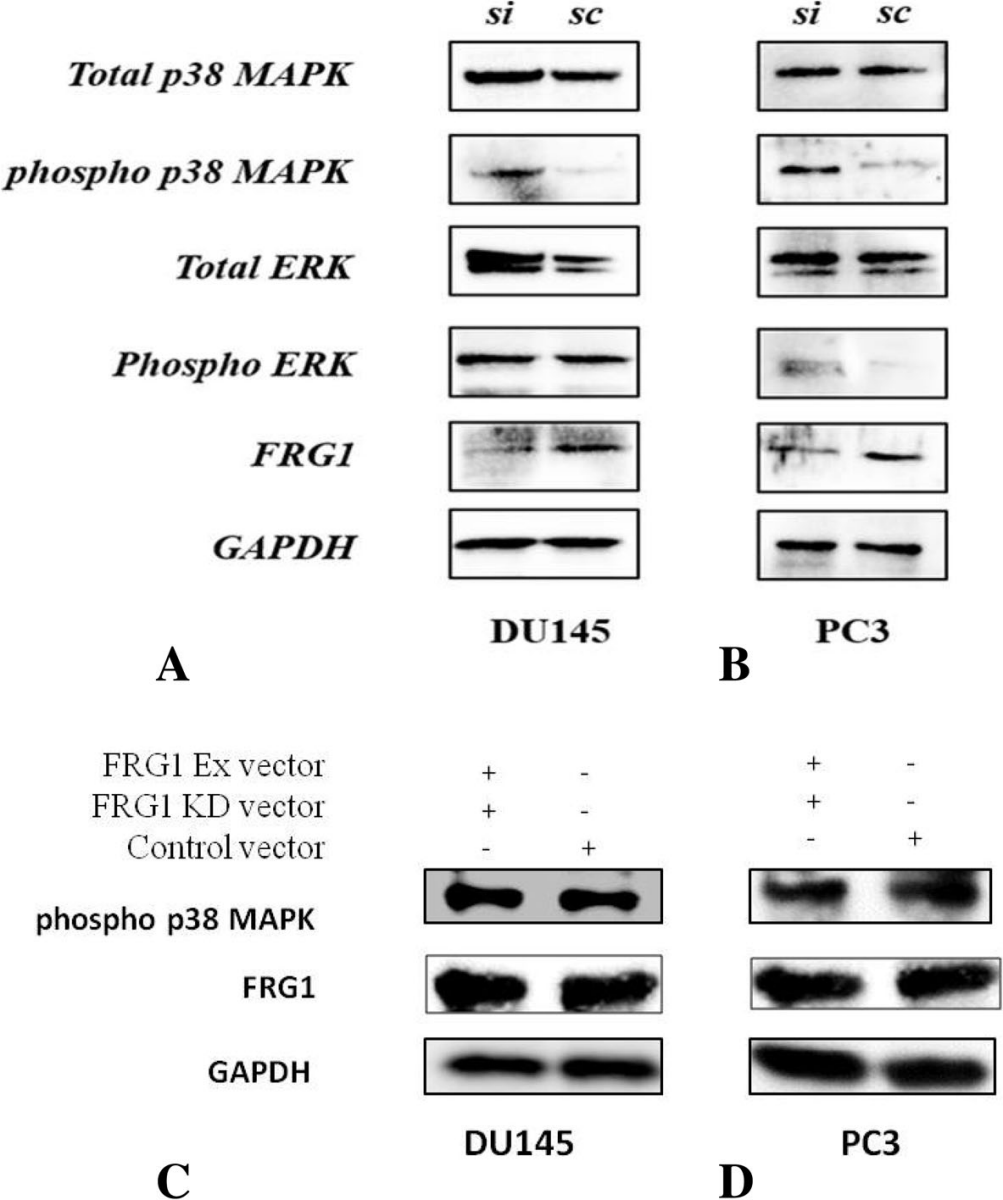
Reduced FRG1 expression enhances p38 MAPK phosphorylation: **a**. Panel of blots showing enhanced p38 MAPK phosphorylation with FRG1 knockdown in DU145 cells but no change in activation of ERK levels. **b**. Panel of blots showing enhanced p38 MAPK phosphorylation with FRG1 knockdown in PC3 cells but mild change in phospho ERK levels. si represents protein lysate from FRG1 knockdown cells and sc represents cells expressing scrambled vector control. **c**. Panel of blot shows neutralization of p38 activation, when FRG1 depletion is rescued by transfection with FRG1 expression vector, in DU145 cells **d**. Panel of blot shows neutralization of p38 activation, when FRG1 depletion is rescued by transfection with FRG1 expression vector, in PC3 cells. All the experiments were repeated three times

To identify effect of FRG1 expression on key signaling pathways we checked the activation levels of ERK and p38 MAPK. FRG1 knockdown showed enhanced phosphorylation of p38 MAPK, in both PC3 and DU145 cells (Fig. 6a-b). On the contrary, no significant difference was observed on ERK phosphorylation levels during FRG1 knockdown in DU145 but slightly elevated levels of phospho ERK were observed in PC3 cells (Fig. 6a-b). Since ERK phosphorylation was neither prominent nor consistent, it was not taken up for further study.

To further confirm the specificity of p38 activation by FRG1 knock down, we transfected FRG1 depleted DU145 and PC3 cells, with FRG1 expression vector. We observed that effect of FRG1 depletion on p38 activation, is rescued in both DU145 (Fig. 6c) and PC3 cells (Fig. 6d).

### FRG1 regulates cytokine expression via p38 MAPK

**Fig. 7:**
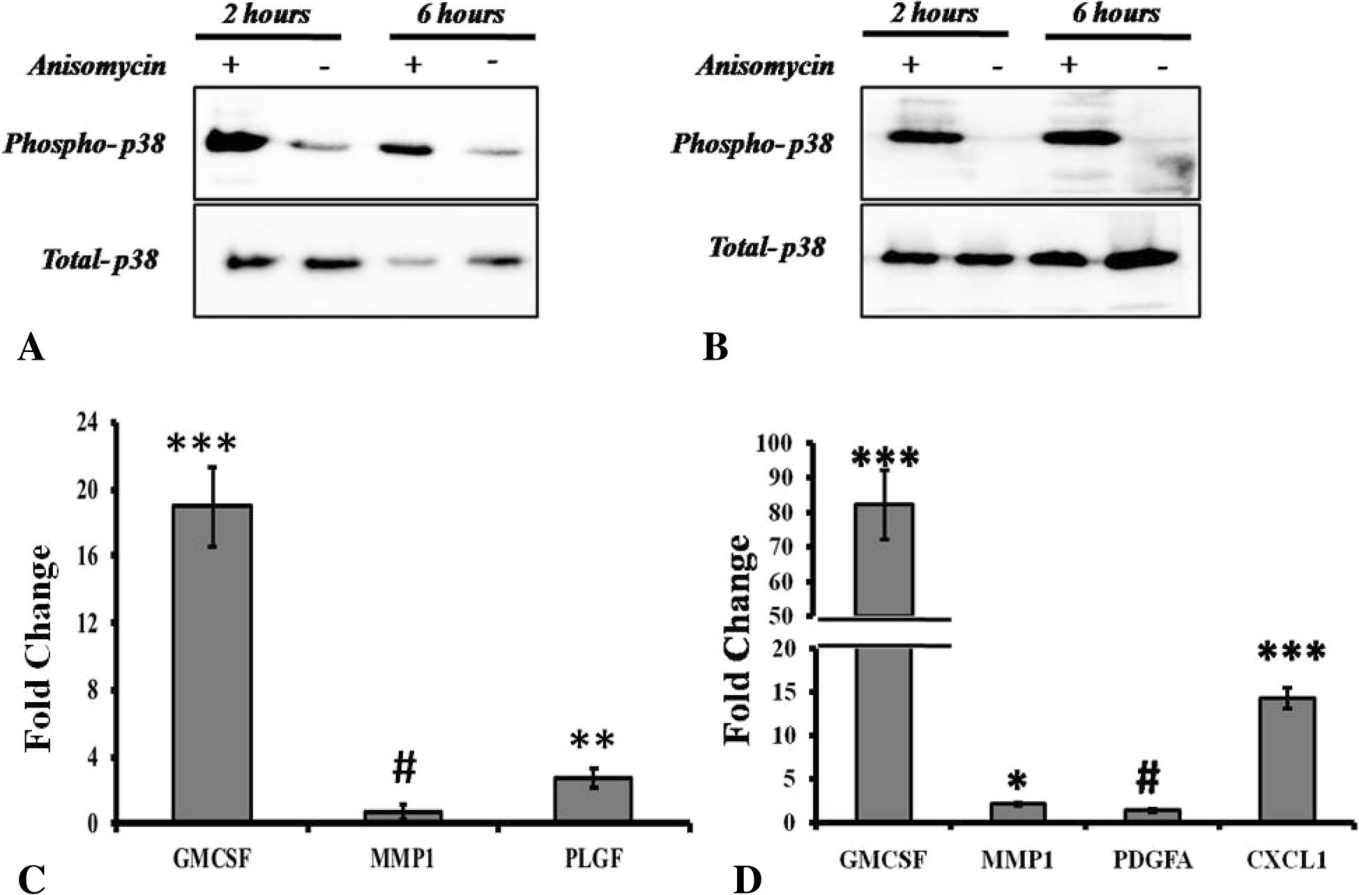
Anisomycin based p38 MAPK activation affects expression of various cytokines and MMPs: **a**. Treatment of the DU145 cells with 0.4 μg/ml of Anisomycin (p38 MAPK activator) led to enhanced phosphorylation of p38 MAPK compared to the mock. Activation state was observed at 2 h (Lane 1 and 2) and 6 h (Lane 3 and 4) time points. **b**. Treatment of the PC3 cells with 0.4 μg/ml of Anisomycin led to enhanced phosphorylation of p38 MAPK compared to the mock. Activation state was observed at 2 h (Lane 1 and 2) and 6 h (Lane 3 and 4). **c**. Expression of GM-CSF, MMP1 and PLGF levels were quantified in DU145 cells after 8 h of treatment with 0.4 μg/ml of Anisomycin. GM-CSF (Fold change = 18.97) and PLGF (Fold change = 2.73) were significantly up-regulated on Anisomycin treatment, no significant effect on MMP1 (Fold change = 0.67) levels were observed. **d**. Expression of GM-CSF, MMP1, PDGFA and CXCL1 levels were quantified in PC3 cells after 8 h of treatment with 0.4 μg/ml of Anisomycin. GM-CSF (Fold change = 82.24), MMP1 (Fold change = 2.07) and CXCL1 (Fold change = 11.98) were significantly up-regulated on Anisomycin treatment, no significant effect on PDGFA (Fold change = 1.36) levels were observed. In panel C and D, t-test (2 tailed, for unpaired samples) was used for comparison of fold change values between experimental and control group. Each experimental group had three replicates. # represents *p* value > 0.05, * represents *p* value < 0.05, ** represents *p* value < 0.01, *** represents *p* value < 0.001

To validate that FRG1 dictates change in expression of cytokines and MMPs (GM-CSF, PLGF and MMP1 in DU145 and, GM-CSF, PDGFA, MMP1 and CXCL1 in PC3 cells), through p38 MAPK, firstly p38 MAPK was activated in DU145 and PC3 cells and, change in expression of previously mentioned cytokines and MMPs was checked by qRT-PCR. In DU145 cells, activation of the p38 MAPK via Anisomycin treatment (Fig. 7a) increased the expression of GM-CSF and PLGF but MMP1 levels were not altered (Fig. 7c). Similarly, in the PC3 cells, p38 MAPK activation (Fig. 7b) increased the expression of GM-CSF, MMP1 and CXCL1 but not of PDGFA (Fig. 7d).

**Fig. 8:**
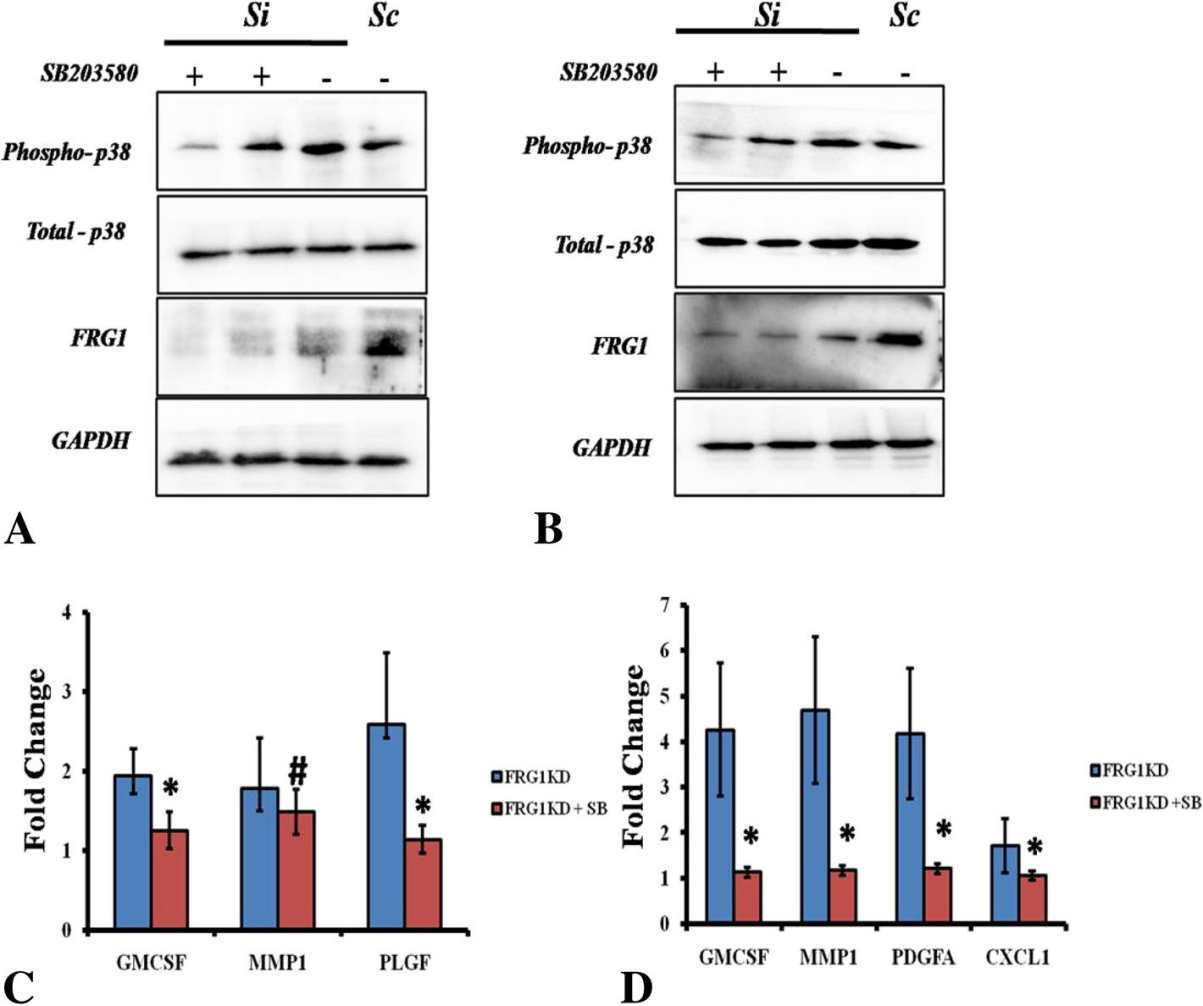
p38 inhibition in FRG1 knockdown prostate cancer cells affects expression of various molecules: **a**. Representative Western blot panel showing reduction of phosphorylated p38 levels in the first lane after treatment with 0.5 μM SB203580 in DU145 FRG1KD cells (Si). Phosphorylated p38 levels were higher in second lane with 0.1 μM SB203580 and in third lane with untreated FRG1KD cells (Si). We could also observe lower p38 phosphorylation levels in DU145 pLKO.1sc (Sc) cells (lane 4) compared to FRG1KD (Si) (lane 3). Third row representing FRG1 panel shows knockdown of FRG1 in FRG1KD (Si) cells compared to pLKO.1 sc (Sc) cells. GAPDH levels were detected as loading control. **b**. Representative western blot panel showing reduction of phosphorylated p38 levels in the first lane after treatment with 0.5 μM SB203580 in PC3 FRG1KD cells (Si). Phosphorylation levels were higher in second lane with 0.1 μM SB203580 and in third lane with untreated FRG1KD cells (Si). We can also observe lower p38 phosphorylation levels in PC3 pLKO.1sc (Sc) cells compared to FRG1KD (Si). Third row representing FRG1 panel shows knockdown of FRG1 in FRG1KD (Si) cells compared to pLKO.1 sc (Sc) cells. GAPDH levels were detected as loading control. **c**. q-RT PCR based expression analysis of GM-CSF, MMP1 and PLGF in DU145 FRG1KD cells after 8 h of treatment with 0.5 μM SB203580 (p38 inhibitor). Fold change in FRG1KD and FRG1KD + SB (p38 inhibitor) groups was derived in comparison with DU145 pLKO.1sc. After 8 h of treatment significant reduction in expression was observed in GM-CSF (FRG1KD, FC = 1.94 vs. FRG1KD + SB, FC = 1.25) and PLGF (FRG1KD, FC = 2.59 vs. FRG1KD + SB, FC = 1.13) expression, no significant reduction was observed in case of MMP1 (FRG1KD, FC = 1.78 to FRG1KD + SB, FC = 1.49). **d**. q-RT PCR based expression analysis of GM-CSF, MMP1, PDGFA and CXCL1 in PC3 FRG1KD cells after 8 h of treatment with 0.5 μM SB203580 (p38 inhibitor). Fold change in FRG1KD and FRG1KD + SB groups was derived in comparison with PC3 pLKO.1sc. After 8 h of treatment significant reduction in GM-CSF (FRG1KD, FC = 4.26 vs. FRG1KD + SB, FC = 1.12), MMP1 (FRG1KD, FC = 4.69 vs. FRG1KD + SB, FC =1.17), PDGFA (FRG1KD, FC = 4.17 vs. FRG1KD + SB, FC = 1.21) and CXCL1 (FRG1KD, FC = 1.71 vs. FRG1KD + SB, FC = 1.05) expression was observed. In panel C and D, t-test (2 tailed, for unpaired samples) was used for comparison of fold change values between experimental and control group. Each experimental group had three replicates. # represents *p* value > 0.05, * represents *p* value < 0.05,, FC represents fold change

For further validation of FRG1’s effect on cytokines and MMPs expression, p38 MAPK was inhibited in DU145 and PC3 cells, with or without FRG1 knockdown. Inhibition of p38 levels in DUI45 cells with FRG1 knockdown, (Fig. 8a) led to the reduction of GM-CSF and PLGF levels but MMP1 levels remained unaltered, compared to DUI45 cells with FRG1 knockdown (Fig. 8c). Inhibition of p38 MAPK in PC3 cells with FRG1 knockdown (Fig. 8b), led to the reduced expression of GM-CSF, MMP1, CXCL1 and PDGFA, compared to PC3 cells with FRG1 knockdown (Fig. 8d). Overall, we found that FRG1 expression levels affect expression of GM-CSF and PLGF in DU145 cell via p38 MAPK. MMP1 expression alteration is not mediated via p38 MAPK. Data in PC3 suggests that FRG1 expression alters, expression of GM-CSF, MMP1 and, CXCL1 via p38 MAPK. Effect on PDGFA expression was not consistent.

These findings suggest that FRG1 might dictate cellular processes through specific cytokines and MMPs via modulation of p38 activity in cell specific manner.

## Discussion

Studies about FRG1 are primarily focused on FSHD pathophysiology and muscle development [17, 18]. Functional studies have shown FRG1 to be actin bundling and RNA binding protein [19, 20], accordingly claiming that FRG1 localizes in both, cytoplasm and nucleus. Our study first time revealed FRG1 expression level and localization in prostate cancer tissue and, showed the significant loss of FRG1 expression in tumor tissues. FRG1 expression was predominantly cytoplasmic but sporadic cases with nuclear localization were also observed. FRG1 expression levels regulate angiogenesis during Xenopus development by affecting *dab2* levels [6]. In our study, no significant association between FRG1 expression and angiogenesis was observed. Involvement of FRG1 in angiogenesis remains unclear as FSHD patients with retinal vasculature abnormalities, showed no change in FRG1 expression [21]. Thus, a well stratified and higher sample size could provide a more conclusive picture regarding localization and role of FRG1 in tumor angiogenesis.

Our study for the first time demonstrates the effect of FRG1 expression on cell properties viz. proliferation, migration and invasion, which are important for tumorigenesis. Prior reports of FRG1 affecting cellular migration, was of myoblast cells in Xenopus development, where FRG1 over-expression enhanced migration and invasion [18]. On the contrary, in our data FRG1 knockdown enhanced cell migration in prostate cancer cells *in vitro*. Which is supported by prior study, where reduced expression of FRG1 was observed in breast cancer cells with higher migratory levels, compared to average non-migratory breast cancer cells [22]. This observation suggests role of FRG1 in migration but in opposite way, indicating that FRG1 function may vary in tumor and developmental set up. Earlier reports have also shown that FRG1 over-expression reduces cell proliferation of mice myoblasts [23]. With varying effect of FRG1 levels in these cell types, an argument can be placed that FRG1 may have discrete effect on various cell types and may dictate cellular properties based on the stromal components.

We found that effect of FRG1 on migration and invasion is also cell type specific. In AR positive LNCaP cells, FRG1 doesn’t effect migration or invasion. This observation can either be attributed to interaction of FRG1 with androgen receptor mediated signaling or to the other differences present in LNCaP cells, compared to DU145 or PC3. Expression pattern of cell surface molecules is similar for both DU145 and PC3 than for LNCaP. DU145 and PC3 cell lines retain cell surface markers such as CD49b/f, CD55 [24] which are reported to have potent role in cancer metastasis [25] [26] [27], through interaction of several pathways, whereas LNCaP cell line lacks these markers, which might be a possible reason for low invasion, migration, proliferation property of LNCaP. Biological function and gene expression is also reported to be similar in DU145 and PC3 [24]. Additionally, LNCaP possesses the differentiated characteristic of prostate secretory cells, in terms of producing prostate-specific antigen and response to androgen regulation; PC3 and DU145 do not show such feature. Moreover, DU145 and PC3 cells are considered to represent more advanced stage of prostate cancer than LNCaP [24] which can justifies more proliferation, invasion, migration property of DU145 and PC3 than LNCaP. Overall, further investigation is required to figure out, why FRG1 expression has no effect in LNCaP cell properties.

FRG1 over-expression had no effect on expression levels of cytokines and MMPs but FRG1 knockdown, in both the prostate cancer cell lines, led to enhanced expression of MMP1, GM-CSF, PLGF, PDGFA and CXCL1 in DU145 and PC3. MMP1 and GM-CSF were up-regulated in both DU145 and PC3 cell lines. FRG1 knockdown led to activation of p38 MAPK; activation of p38 MAPK has been associated with tumor progression previously [28]. In prostate cancer p38 MAPK activation has been reported via TNFα and IL6 [28, 29]. We are first time reporting the involvement of FRG1 in p38 MAPK mediated signaling, which can be very well connected with our data on various cytokines expression and explains the results of cell based assays. We found that GM-CSF was up-regulated in both DU145 and PC3 cell lines, with reduced FRG1 expression; earlier study has shown that both these cell lines are positive for GM-CSF receptor [30]. Treatment of DU145 and PC3 cells with GM-CSF has been shown to enhance colonogenicity and chemotaxis [30]. Up-regulation of GM-CSF with FRG1 knockdown in prostate cancer cells might be one of the factors affecting cell migration and invasiveness. Prior studies in human monocytes and bronchial epithelial cells have observed that p38 MAPK activation regulates GM-CSF production [31]. In this study, we have clearly shown that GM-CSF levels are altered with activation and inhibition of p38 MAPK. CXCL1 is known to enhance tumor stromal interaction leading to enhanced migration and invasion in various cancer [32]. With respect to prostate cancer, CXCL1 is known to affect cell migration and invasion via NFĸB/HDAC [33]. TNF alpha enhances p38 phosphorylation in endothelial cells which up-regulates CXCL1 levels (20). Here we report that FRG1 knockdown leads to p38 activation in prostate cancer cells affecting CXCL1 expression, which is well supported by previous studies, except the role of FRG1. Activation of p38 MAPK also induces expression of MMP1, promoting invasiveness in cell lines [34, 35]. But according to our finding MMP1 expression varies with cell type, suggesting that MMP1 levels might be regulated by FRG1 independent of p38 MAPK activity. PDGFA signaling is known to activate p38 MAPK in porcine aortic endothelial cells, leading to enhanced cellular migration [31]. Prior reports suggest that PC3 cells but not DU145, is positive for PDGFA receptor [36] which can be associated with activated p38 MAPK via enhanced PDGFA expression. Our findings show that p38 activation doesn’t affect PDGFA levels but inhibition of p38 MAPK showed reduced expression of PDGFA. Similar to PDGF signaling, PLGF also activates p38 MAPK and, is known to enhance cellular migration in leukemia [37]. PLGF binds to Flt 1 receptor and exert activation of p38 MAPK in DU145 cells [38]. We have also first time reported effect on PLGF expression directly under the influence of p38 activation.

**Fig. 9:**
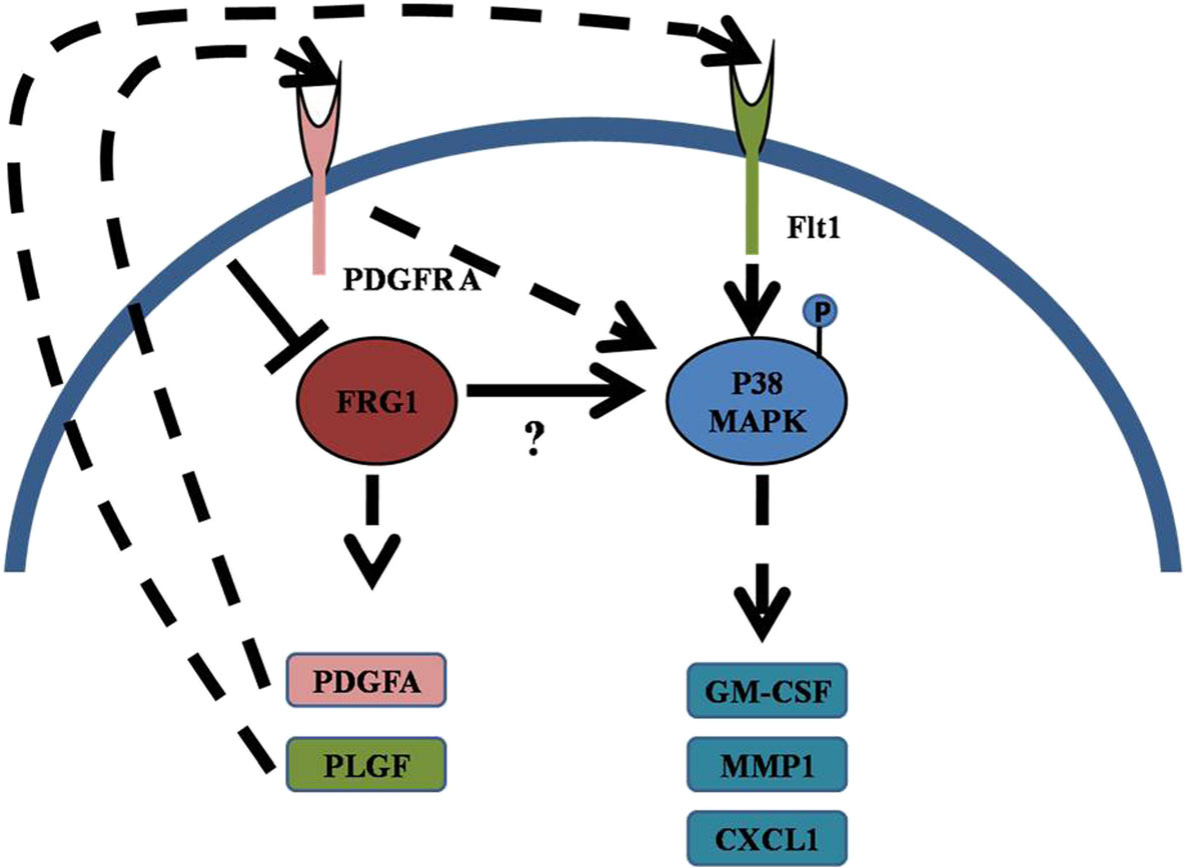
Representation model for possible molecular interaction during FRG1 knockdown in prostate cancer cells

Overall, the effect exerted by FRG1 on prostate cancer cell properties and expression of various cytokines is mediated via p38 MAPK activity (Fig. 9). However, there is an impending question, regarding the mechanism of p38 activation during FRG1 knockdown. With no prior reports of association of FRG1 with above-mentioned molecules, which regulate tumor progression, further mechanistic studies are required to establish how FRG1 activates p38 MAPK, eventually leading to expression of certain cytokines and MMPs.

## Conclusion

FRG1 expression levels are reduced in prostate tumor tissue and its expression affects the cell migratory and invasiveness properties of AR negative prostate cancer cell lines. FRG1 may exert its effect on tumorigenic properties through specific cytokines and MMPs via p38 MAPK activation. However, a better understanding is required to know on how FRG1 regulates these molecules.

## Additional files

Additional file 1: **Table S1.** Genes with list of primers for which expression was determined in DU145 and PC3 cells with altered FRG1 expression. (PDF 37 kb)

Additional file 2: **Table S2.** Prostate cancer cohort used for IHC analysis of FRG1 expression. (PDF 118 kb)

Additional file 3: Correlation analysis of tumor IRS for FRG1, with Gleason score and MVD count. (PDF 96 kb)

Additional file 4: q-RT PCR based analysis of gene expression in DU145 cells and PC3 cells, with ectopic expression of FRG1. (PDF 104 kb)

## Abbreviations

BMP4: Bone Morphogenetic Protein 4
CXCL1: Chemokine (C-X-C motif) ligand 1
dab2: Disabled 2
FRG1: FSHD Region Gene 1
FSHD: Facioscapulohumeral Muscular Dystrophy
GM-CSF: Granulocyte Macrophage colony stimulating factor
HRP: Horse Radish Peroxidase
mDEC6: Murine Dental Epithelial Cell 6
MMP: Matrix metalloproteinase
MVD: Micro Vessel Density
NCCS: National Centre for Cell Science
PDGFA: Platelet Derived Growth Factor A
PDGFRα: Platelet Derived Growth Factor Receptor α
PLGF: Placental Growth Factor
