# Supplementary material for: Reduced FRG1 expression promotes prostate cancer progression and affects prostate cancer cell migration and invasion
Source: BMC Cancer. 2019 Apr 11;19:346. doi: 10.1186/s12885-019-5509-4 (PMC6458714; doi:10.1186/s12885-019-5509-4)
Supplement: Supplementary file 1 — Table S1. Genes with list of primers for which expression was determined in DU145 and PC3 cells with altered FRG1 expression. (PDF 37 kb) [file 12885_2019_5509_MOESM1_ESM.pdf]

Table S1: Genes with list of primers for which expression was determined in DU145 and PC3 cells with altered FRG1 expression

Gene	Primer 5`---- 3`
MMP1 F	AGAGCAGATGTGGACCATGC
MMP1 R	TTGTCCCGATGATCTCCCCT
MMP2 F	CGTCGCCCATCATCAAGTTC
MMP2 R	CAGGTATTGCACTGCCAACTC
MMP3 F	CACTCACAGACCTGACTCGG
MMP3 R	AGTCAGGGGGAGGTCCATAG
MMP8 F	AAGCCAGGAGGGGTAGAGTT
MMP8 R	TTTTCCAGGTAGTCCTGAACAGT
MMP9 F	TTCAGGGAGACGCCCATTTC
MMP9 R	AACCGAGTTGGAACCACGAC
MMP10 F	AGTTTGGCTCATGCCTACCC
MMP10 R	TTGGTGCCTGATGCATCTTCT
MMP13 F	GTTTGCAGAGCGCTACCTGA
MMP13 R	GACTGCATTTCTCGGAGCCT
FGF2 F	GCTGTACTGCAAAAACGGGG
FGF2 R	TAGCTTGATGTGAGGGTCGC
PLGF F	CCATGCAGCTCCTAAAGATCC
PLGF R	TCCTCCTTTCCGGCTTCA
CXCL1 F	AACCGAAGTCATAGCCACAC
CXCL1 R	GTTGGATTTGTCACTGTTCAGC
CXCL8 F	ACCGGAAGGAACCATCTCAC
CXCL8 R	GGCAAAACTGCACCTTCACAC
IL 10 F	AAGACCCAGACATCAAGGCG
IL 10 R	AATCGATGACAGCGCCGTAG
PDGFA F	GCCAACCAGATGTGAGGTGA
PDGFA R	GGAGGAGAACAAAGACCGCA
PDGFB F	ACCTGCGTCTGGTCAGC
PDGFB R	ATCTTCCTCTCCGGGGTCTC
GM-CSF F	CTGGAGCTGTACAAGCAGGG
GM-CSF R	ACAGGAAGTTTCCGGGGTTG
G-CSF F	AGCAAGTGAGGAAGATCCAGG
G-CSF R	TTGTAGGTGGCACACTCACTC
VEGFA-F	ATCTGCATGGTGATGTTGGA
VEGFA-R	GGGCAGAATCATCACGAAGT
TGF-beta-F	GCAACAATTCCTGGCGATACC
TGF-beta-R	AAAGCCTCAATTTCCCCTCC
