# Supplementary material for: Reduced FRG1 expression promotes prostate cancer progression and affects prostate cancer cell migration and invasion
Source: BMC Cancer. 2019 Apr 11;19:346. doi: 10.1186/s12885-019-5509-4 (PMC6458714; doi:10.1186/s12885-019-5509-4)
Supplement: Supplementary file 2 — Table S2. Prostate cancer cohort used for IHC analysis of FRG1 expression. (PDF 118 kb) [file 12885_2019_5509_MOESM2_ESM.pdf]

Table S2: Prostate cancer cohort used for IHC analysis of FRG1 expression.

S. No.	Age Range* (years)	Organ/Anatomic Site	Pathology diagnosis	Type	Gleason Score
1	71 or over	Prostate	Adenocarcinoma	Malignant	2+3=5
2	71 or over	Prostate	Adenocarcinoma	Malignant	3+2=5
3	61-70	Prostate	Adenocarcinoma	Malignant	3+3=6
4	71 or over	Prostate	Adenocarcinoma	Malignant	3+3=6
5	71 or over	Prostate	Adenocarcinoma	Malignant	3+3=6
6	71 or over	Prostate	Adenocarcinoma	Malignant	3+3=6
7	61-70	Prostate	Adenocarcinoma	Malignant	3+3=6
8	71 or over	Prostate	Adenocarcinoma	Malignant	3+3=6
9	71 or over	Prostate	Adenocarcinoma	Malignant	3+3=6
10	71 or over	Prostate	Adenocarcinoma	Malignant	3+3=6
11	71 or over	Prostate	Adenocarcinoma	Malignant	3+3=6
12	61-70	Prostate	Adenocarcinoma	Malignant	3+3=6
13	61-70	Prostate	Adenocarcinoma	Malignant	3+3=6
14	61-70	Prostate	Adenocarcinoma	Malignant	3+3=6
15	71 or over	Prostate	Adenocarcinoma	Malignant	3+3=6
16	61-70	Prostate	Adenocarcinoma	Malignant	3+3=6
17	71 or over	Prostate	Adenocarcinoma	Malignant	3+3=6
18	61-70	Prostate	Adenocarcinoma	Malignant	3+3=6
19	71 or over	Prostate	Adenocarcinoma	Malignant	3+3=6
20	71 or over	Prostate	Adenocarcinoma	Malignant	3+3=6
21	71 or over	Prostate	Adenocarcinoma	Malignant	3+3=6
22	71 or over	Prostate	Adenocarcinoma	Malignant	3+3=6
23	61-70	Prostate	Adenocarcinoma	Malignant	3+3=6
24	71 or over	Prostate	Adenocarcinoma	Malignant	3+3=6
25	61-70	Prostate	Adenocarcinoma	Malignant	3+3=6
26	71 or over	Prostate	Adenocarcinoma	Malignant	3+3=6
27	71 or over	Prostate	Adenocarcinoma	Malignant	3+3=6
28	61-70	Prostate	Adenocarcinoma	Malignant	3+3=6
29	61-70	Prostate	Adenocarcinoma	Malignant	3+3=6
30	71 or over	Prostate	Adenocarcinoma	Malignant	3+4=7
31	71 or over	Prostate	Adenocarcinoma	Malignant	3+4=7
32	71 or over	Prostate	Adenocarcinoma	Malignant	3+4=7
33	61-70	Prostate	Adenocarcinoma	Malignant	3+4=7
34	61-70	Prostate	Adenocarcinoma	Malignant	3+4=7
35	51-60	Prostate	Adenocarcinoma	Malignant	3+4=7
36	51-60	Prostate	Adenocarcinoma	Malignant	3+4=7
37	71 or over	Prostate	Adenocarcinoma	Malignant	3+4=7
38	71 or over	Prostate	Adenocarcinoma	Malignant	3+4=7
39	61-70	Prostate	Adenocarcinoma	Malignant	3+4=7
40	61-70	Prostate	Adenocarcinoma	Malignant	3+4=7
41	61-70	Prostate	Adenocarcinoma	Malignant	3+4=7
42	71 or over	Prostate	Adenocarcinoma	Malignant	3+4=7
43	71 or over	Prostate	Adenocarcinoma	Malignant	3+4=7
44	51-60	Prostate	Adenocarcinoma	Malignant	3+4=7
45	71 or over	Prostate	Adenocarcinoma	Malignant	3+4=7
46	61-70	Prostate	Adenocarcinoma	Malignant	3+4=7
47	71 or over	Prostate	Adenocarcinoma	Malignant	3+4=7
48	61-70	Prostate	Adenocarcinoma	Malignant	3+5=8
49	61-70	Prostate	Adenocarcinoma	Malignant	3+5=8
50	71 or over	Prostate	Adenocarcinoma	Malignant	3+5=8

51	61-70	Prostate	Adenocarcinoma	Malignant	4+3=7
52	71 or over	Prostate	Adenocarcinoma	Malignant	4+3=7
53	61-70	Prostate	Adenocarcinoma	Malignant	4+3=7
54	51-60	Prostate	Adenocarcinoma	Malignant	4+3=7
55	61-70	Prostate	Adenocarcinoma	Malignant	4+3=7
56	71 or over	Prostate	Adenocarcinoma	Malignant	4+3=7
57	51-60	Prostate	Adenocarcinoma	Malignant	4+3=7
58	71 or over	Prostate	Adenocarcinoma	Malignant	4+3=7
59	61-70	Prostate	Adenocarcinoma	Malignant	4+3=7
60	61-70	Prostate	Adenocarcinoma	Malignant	4+3=7
61	61-70	Prostate	Adenocarcinoma	Malignant	4+3=7
62	61-70	Prostate	Adenocarcinoma	Malignant	4+3=7
63	71 or over	Prostate	Adenocarcinoma	Malignant	4+3=7
64	61-70	Prostate	Adenocarcinoma	Malignant	4+3=7
65	61-70	Prostate	Adenocarcinoma	Malignant	4+3=7
66	71 or over	Prostate	Adenocarcinoma	Malignant	4+3=7
67	61-70	Prostate	Adenocarcinoma	Malignant	4+4=8
68	61-70	Prostate	Adenocarcinoma	Malignant	4+4=8
69	71 or over	Prostate	Adenocarcinoma	Malignant	4+4=8
70	71 or over	Prostate	Adenocarcinoma	Malignant	4+4=8
71	61-70	Prostate	Adenocarcinoma	Malignant	4+4=8
72	51-60	Prostate	Adenocarcinoma	Malignant	4+4=8
73	61-70	Prostate	Adenocarcinoma	Malignant	4+4=8
74	51-60	Prostate	Adenocarcinoma	Malignant	4+4=8
75	71 or over	Prostate	Adenocarcinoma	Malignant	4+4=8
76	61-70	Prostate	Adenocarcinoma	Malignant	4+4=8
77	61-70	Prostate	Adenocarcinoma	Malignant	4+5=9
78	51-60	Prostate	Adenocarcinoma	Malignant	4+5=9
79	71 or over	Prostate	Adenocarcinoma	Malignant	4+5=9
80	71 or over	Prostate	Adenocarcinoma	Malignant	4+5=9
81	71 or over	Prostate	Adenocarcinoma	Malignant	4+5=9
82	71 or over	Prostate	Adenocarcinoma	Malignant	4+5=9
83	61-70	Prostate	Adenocarcinoma	Malignant	5+4=9
84	71 or over	Prostate	Adenocarcinoma	Malignant	5+4=9
85	61-70	Prostate	Adenocarcinoma	Malignant	5+4=9
86	71 or over	Prostate	Adenocarcinoma	Malignant	5+4=9
87	61-70	Prostate	Adenocarcinoma	Malignant	5+4=9
88	71 or over	Prostate	Adenocarcinoma	Malignant	5+4=9
89	71 or over	Prostate	Adenocarcinoma	Malignant	5+4=9
90	61-70	Prostate	Adenocarcinoma	Malignant	5+5=10
91	Not Available	Prostate	Adenocarcinoma	Malignant	4+4=8
92	Not Available	Prostate	Adenocarcinoma	Malignant	5+4=9
93	Not Available	Prostate	Adenocarcinoma	Malignant	4+4=8
94	Not Available	Prostate	Adenocarcinoma	Malignant	3+3=6
95	Not Available	Prostate	Adenocarcinoma	Malignant	3+3=6
96	Not Available	Prostate	Adenocarcinoma	Malignant	5+5=10
97	Not Available	Prostate	Adenocarcinoma	Malignant	3+4=7
98	Not Available	Prostate	Adenocarcinoma	Malignant	5+4=9
99	Not Available	Prostate	Adenocarcinoma	Malignant	5+5=10
100	Not Available	Prostate	Adenocarcinoma	Malignant	4+4=8

*** Age range 51-60, 61- 70, 71 or over**
