# Supplementary material for: Reduced FRG1 expression promotes prostate cancer progression and affects prostate cancer cell migration and invasion
Source: BMC Cancer. 2019 Apr 11;19:346. doi: 10.1186/s12885-019-5509-4 (PMC6458714; doi:10.1186/s12885-019-5509-4)
Supplement: Supplementary file 3 — Correlation analysis of tumor IRS for FRG1, with Gleason score and MVD count. (PDF 96 kb) [file 12885_2019_5509_MOESM3_ESM.pdf]

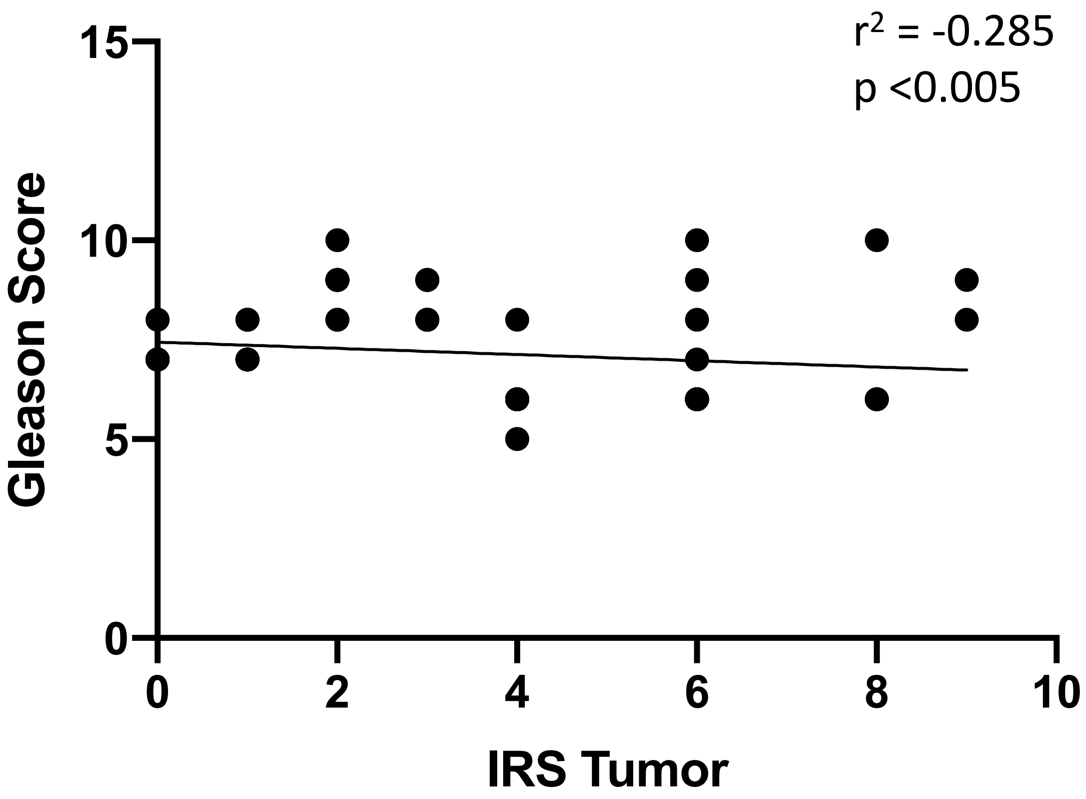

Graphical Representation of Correlation analysis between Tumor IRS for FRG1 and Gleason Score

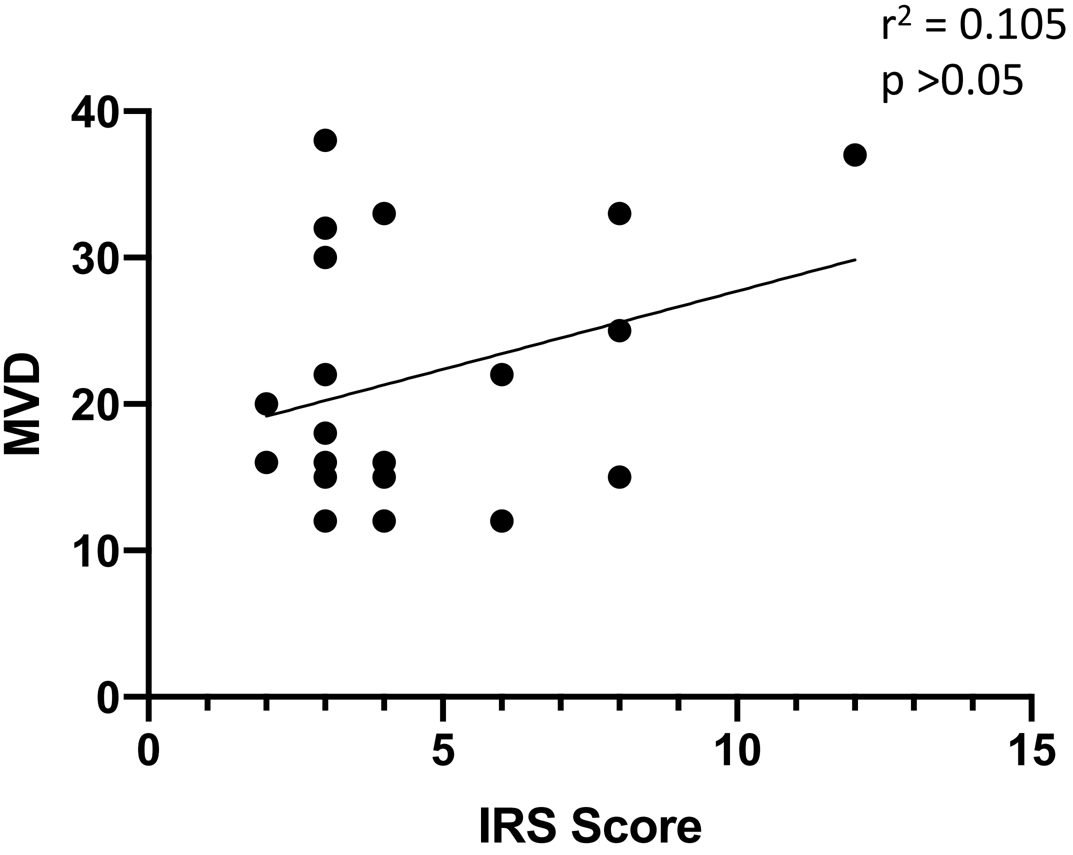

Graphical Representation of Correlation analysis between IRS and MVD count
