# Supplementary material for: Reduced FRG1 expression promotes prostate cancer progression and affects prostate cancer cell migration and invasion
Source: BMC Cancer. 2019 Apr 11;19:346. doi: 10.1186/s12885-019-5509-4 (PMC6458714; doi:10.1186/s12885-019-5509-4)
Supplement: Supplementary file 4 — q-RT PCR based analysis of gene expression in DU145 cells and PC3 cells, with ectopic expression of FRG1. (PDF 104 kb) [file 12885_2019_5509_MOESM4_ESM.pdf]

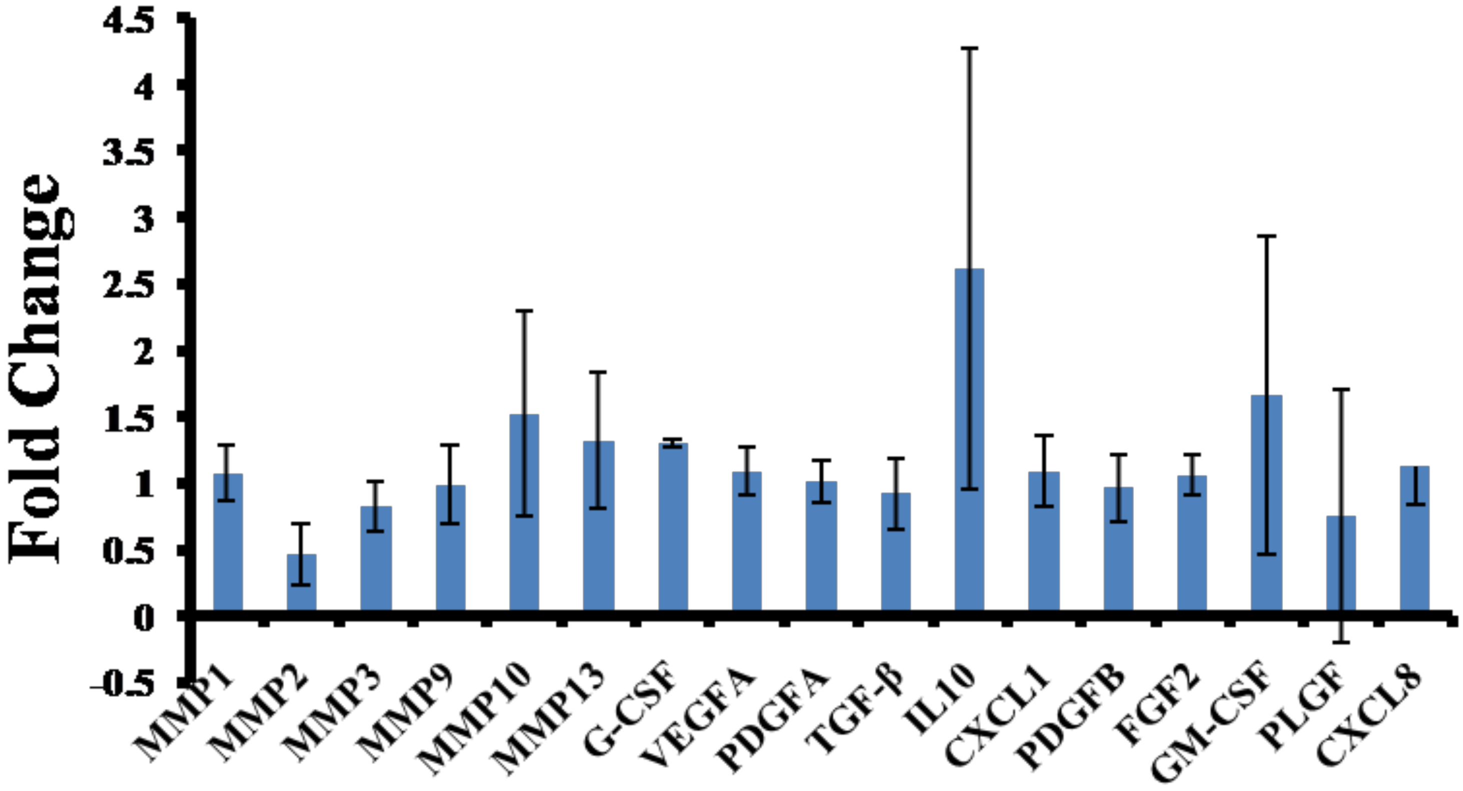

q-RT PCR based analysis of gene expression in DU145 cells with ectopic expression of FRG1, compared to empty vector control. X-axis shows the names of genes analyzed and Y-axis shows fold change.

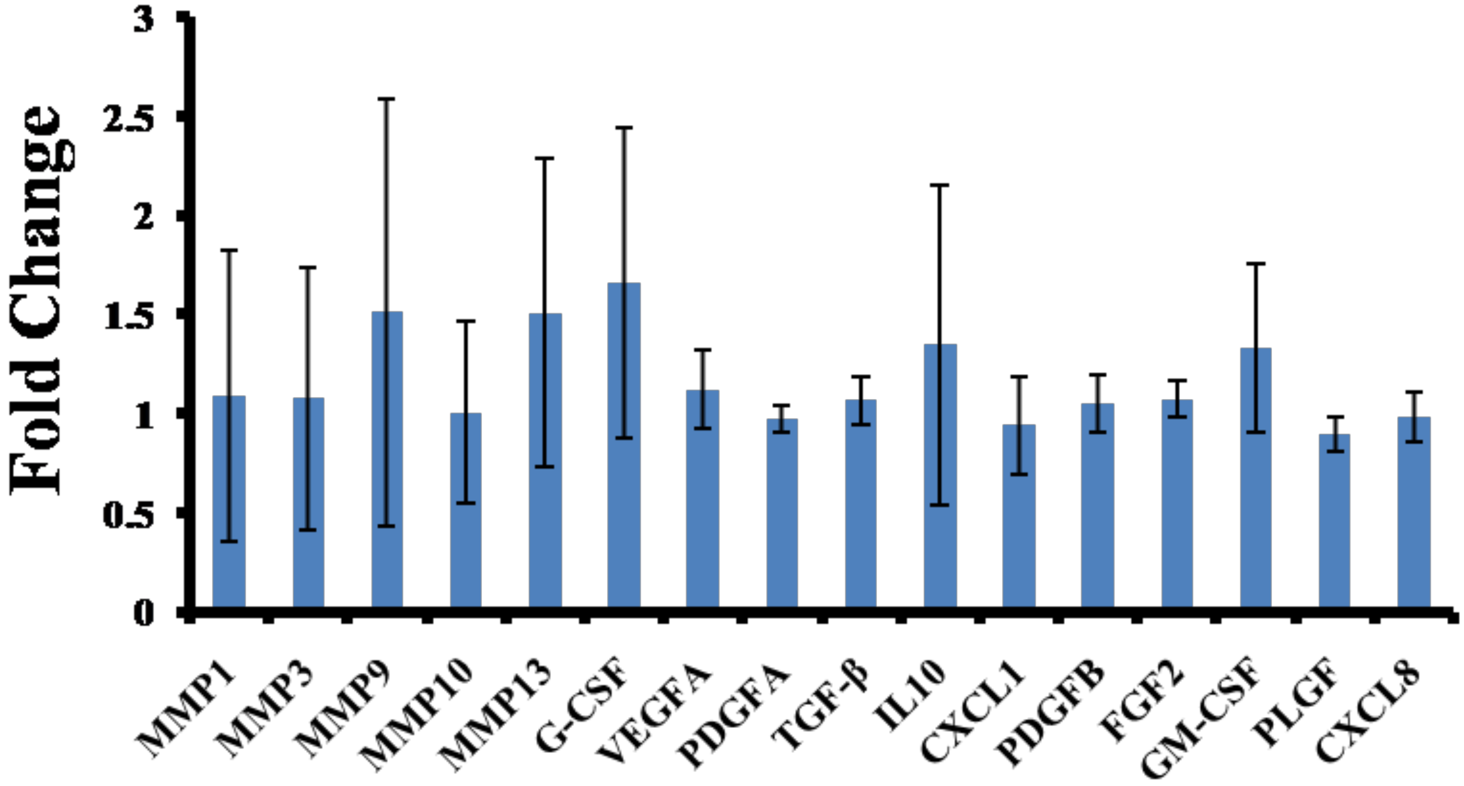

q-RT PCR based analysis of gene expression in PC3 cells with ectopic expression of FRG1, compared to empty vector control. X-axis shows the names of genes analyzed and Y-axis shows fold change.
